# Indicated Prevention of Fetal Alcohol Spectrum Disorders in South Africa: Effectiveness of Case Management

**DOI:** 10.3390/ijerph13010076

**Published:** 2015-12-23

**Authors:** Marlene M. de Vries, Belinda Joubert, Marise Cloete, Sumien Roux, Beth A. Baca, Julie M. Hasken, Ronel Barnard, David Buckley, Wendy O. Kalberg, Cudore L. Snell, Anna-Susan Marais, Soraya Seedat, Charles D. H. Parry, Philip A. May

**Affiliations:** 1 Department of Psychiatry, Faculty of Medicine and Health Sciences, Stellenbosch University, Tygerberg 7505, South Africa; bjoubert@sun.ac.za (B.J.); lcloete@sun.ac.za (M.C.); sumien@sun.ac.za (S.R.); ronelb07@gmail.com (R.B.); asmarais@sun.ac.za (A.-S.M.); sseedat@sun.ac.za (S.S.); cparry@sun.ac.za (C.D.H.P); 2 Center on Alcoholism, Substance Abuse, and Additions (CASAA), The University of New Mexico, Albuquerque, NM 87106, USA; babaca@unm.edu (B.A.B.); dbuckley@unm.edu (D.B.); wkalberg@unm.edu (W.O.K.); 3 Gillings School of Global Public Health, Department of Nutrition, Nutrition Research Institute, The University of North Carolina at Chapel Hill, Kannapolis, NC 28081, USA; julie_hasken@unc.edu; 4 School of Social Work, Howard University, Washington, DC 20059, USA; csnell@howard.edu; 5 Tobacco & Other Drug Research Unit, Medical Research Council of South Africa, Alcohol, Tygerberg 7505, South Africa

**Keywords:** alcohol exposure, case management, pregnancy, happiness, fetal alcohol spectrum disorders, South Africa, reduction in alcohol consumption

## Abstract

In the Western Cape Province of South Africa (ZA) a subculture of binge drinking produces the highest global documented prevalence of fetal alcohol spectrum disorders (FASD). FASD prevention research activities in ZA use the Comprehensive Prevention approach from the United States Institute of Medicine. Case management (CM) was delivered as a method of indicated prevention to empower heavy drinking pregnant women to achieve cessation or a reduction in drinking. CM activities incorporated life management, Motivational Interviewing (MI) techniques and the Community Reinforcement Approach (CRA). Data were collected at baseline, 6, 12 and 18 months. Mean drinking decreases 6 months into CM; but overall alcohol consumption rose significantly over time to levels higher than baseline at 12 and 18 months. Alcohol consumption drops significantly from before pregnancy to the second and third trimesters. AUDIT scores indicate that problematic drinking decreases significantly even after the vulnerable fetus/baby was born. CM significantly increases client happiness, which correlates with reduced weekend drinking. CM was successful for women with high-risk drinking behaviour, and was effective in helping women stop drinking, or drink less, while pregnant, reducing the risk of FASD.

## 1. Introduction

Abstinence from alcohol during pregnancy is an important and theoretically attainable goal with known benefits for the unborn child. Alcohol exposure during pregnancy can cause fetal alcohol spectrum disorders (FASD), which are the leading known preventable forms of birth defects and developmental disabilities [1,2]. FASD include fetal alcohol syndrome (FAS) and partial fetal alcohol syndrome (PFAS), which are characterized by a unique pattern of facial features, physical growth deficits, and neurobehavioral deficits, as well as alcohol-related neurodevelopmental disorders (ARND) characterized expressly by a unique set of neurobehavioral deficits [1]. These alcohol-related birth defects result from prenatal alcohol exposure and the interaction with maternal risk factors such as age, childbearing history, physical size, and nutrition [3,4]. In order to prevent all cases of FASD, abstinence is best, for there is no safe level of alcohol that can be consumed by pregnant women [5,6]. Therefore, this intervention study aimed to eliminate, or at least reduce, alcohol use in pregnant women in South Africa (ZA) through one-on-one case management (CM).

The prevalence of FASD in the Western Cape Province of ZA is the highest documented in the world (136 to 209 per 1000 children or 13.6% to 20.9%) [7]. In this region, a subculture of binge drinking makes it common for up to 40% of women of childbearing age to drink two to nine alcoholic beverages each Friday and Saturday night [8,9,10,11]. The economy of the Western Cape Province is to a large extent based on grape growing for wine production and the production of deciduous fruits. Since many people in the study population have low levels of education, their livelihood depends on either farm work or manual labor. With wine and fruit production making use of seasonal labor, people in the study population often have a low and irregular income, leaving many households with insufficient resources. Housing is scarce and informal settlements without basic services as well as overpopulation are common. These circumstances contribute to social problems such as alcohol and drug abuse, family violence and a circle of poverty preventing people from reaching their full potential. Weekend drinking is institutionalized and is seen as a normal way of life and a valued form of recreation [8,9,10]. Drinking is not only a social affair and a regular source of relaxation, but also a way of coping with unpleasant realities. Given the high rates of weekend binge drinking during pregnancy and the resultant high rates of FASD, specific subpopulations of women in the Western Cape were targeted for an indicated prevention model of CM.

CM was undertaken as one component of a community-wide, comprehensive prevention program developed from the United States Institute of Medicine (IOM) model [2]. The IOM Comprehensive prevention model targets prevention at three levels: universal, selected, and indicated. Universal methods focus on public education and policy aimed at the entire population, selected prevention targets women of childbearing age with a focus on avoiding alcohol before and during pregnancy, and indicated prevention is a tertiary level approach for women who are already pregnant and drink large amounts of alcohol [2]. CM is a behavioral intervention and consists of a set of social service functions focused on education, coaching, and support to help women assess their inner strengths and external resources in order to change their drinking behavior. 

In this study, CM aimed to support heavy drinking pregnant women to achieve cessation or a reduction in drinking for the benefit of their baby both during pregnancy and in the first year of life. The primary goal was to protect the health of the fetus through prenatal care by motivating and supporting women, with the ultimate goal of reducing the prevalence of FASD. A secondary goal was to understand the association between drinking behaviour and happiness during CM. Results for the first 41 women who began CM were previously reported [12]. This paper builds on those data with the inclusion of an additional 26 individuals enrolled in CM, of which 18 completed CM. The analyses reported in this paper provide an update and further analysis of the drinking data previously reported, which serve as a meaningful backdrop for better understanding the association between drinking and happiness while in CM.

## 2. Experimental Procedures

### 2.1. Methods

This prospective, intervention study was carried out at community health clinics in the Western Cape province of ZA between January 2009 and June 2011. Program staff recruited women at high risk for bearing a child with FASD to participate in an 18-month case management intervention aimed at reducing alcohol consumption. Women were screened for study eligibility, and CM started as soon as possible after women were deemed eligible for the study. The program staff collected data from the participants at the time of their screening and at a baseline visit that coincided with the start of case management. The program staff collected similar data at 6, 12, and 18-month intervals during the CM intervention. Data collection included measures of weekend drinking and problematic drinking behaviors as well as the Happiness Scale, which is a measure of well-being and contentment. 

### 2.2. Sample and Recruitment

Women were recruited from antenatal community health clinics as early as possible in pregnancy and, once enrolled, followed for 18 months. A total of 67 pregnant women enrolled in CM. Most women began CM in their second trimester of pregnancy (*n* = 41, 61.2%), but thirteen were in their first trimester (19.4%) and 13 began CM in their third trimester (19.4%). Historically, women in ZA go to the antenatal clinics for pregnancy testing around 20 weeks gestation or later and do not acknowledge their pregnancy until a positive pregnancy test is confirmed by the antenatal nurse. Most ZA women consider this confirmation of pregnancy as the beginning of their pregnancy. 

The intervention was targeted at women at high risk for bearing a child with FASD. Field workers invited all pregnant women visiting the community health clinics to participate in a screening which included the Self-Administered Questionnaire (SAQ) [13] and the Alcohol Use Disorders Identification Test (AUDIT) [14]. The SAQ and AUDIT provide a measure of prenatal alcohol use. These screening instruments were used to determine women with high risk drinking behavior and other risk factors contributing to a potential child with FASD. Specifically, women were recruited if they met one or more of the following criteria: (1) had a child previously diagnosed with FASD; (2) drank heavily during a previous pregnancy; (3) had eight or more drinks per week during the current pregnancy or had one binge of three or more drinks any day of the week; or (4) had a score of 8 or more on the AUDIT or a score of 2 or more on the SAQ, which is consistent with heavy drinking [13,14]. Women who met these criteria were invited to participate in CM on a regular basis for 18 months. Women who did not meet any of the above inclusion criteria were excluded from the study. 

CM was initiated with women as early as possible after the screening in order to reduce drinking behaviors early in the pregnancy. The recruitment procedures and CM process were described in detail in a previous publication [12]. Overall retention was good, with 76% of these women (*n* = 51) completing the 18 month program. For the 16 women who dropped out of CM, 6 women dropped out by 6 months, six women dropped out between 6 and 12 months, and an additional four dropped out between 12 and 18 months. Women dropped out of the study for a number of reasons, including relocation out of the area and limited time when there was seasonal work. 

Active consent for subjects to participate in all phases of this study of CM was obtained. Protocols and consent forms were approved by The University of New Mexico School of Medicine, HRRC # 96–209, and Stellenbosch Faculty of Health Sciences Ethics Committee.

### 2.3. Case Management Model

CM helps women set goals and assess the resources, both internal and external, that are needed to reduce their drinking or abstain from alcohol during pregnancy [15,16,17]. Home visits and/or visits to the office were completed on a monthly basis and could, depending on the circumstances and needs of the women in CM, be extended to twice a month. Many of these visits occurred during the lunch hour if the women were employed, since these women could not afford to lose a day’s wages in order to schedule an appointment with the case manager. Women were able to choose the best location for their visits, whether it was at home or at the field office, and each visit lasted approximately 30 min. In addition to the in-person visits, telephone contact was initiated by the case manager once or twice per month. Women in CM were also given a cell phone number that they could send “please-call-me” messages when they needed support from their case manager. 

Field staff used proven principles and methods of social work, motivational interviewing (MI) and community reinforcement approach (CRA) to encourage positive changes in lifestyle, childbearing practices and drinking behavior. MI is a collaborative, person-centered form of guiding and counseling that elicits and strengthens motivation for change while being respectful, quietly attentive, and supportive of an individual’s right to make decisions and take action. MI is based on four primary principles: (1) expressing empathy through reflective listening; (2) developing discrepancy in clients about negative impact of current behavior on important goals and values; (3) navigating client resistance in order to avoid arguments that undermine changes; and (4) supporting self-efficacy by expressing optimism for change, avoiding the role of expert and highlighting a client’s responsibility to choose and carry out changes [18,19]. CRA is a comprehensive behavioral program for treating substance abuse problems aimed at making a sober lifestyle more rewarding than the use of substances [20,21]. During the CM process, case managers also made use of timelines, models of the fetus at 3–4 months gestation to demonstrate size, and development charts of fetuses demonstrating growth and development. Additionally, case managers used ecocharts or ecomaps as a tool to illustrate and identify family, friends, and organizations that could play a supportive role in the goal of reduced drinking [22]. 

Field staff (social workers and nurses) received two weeks of intensive and specialized training by experts in social work, MI, CRA, and prevention. Most of the training was completed in Afrikaans, the first language of the field staff as well as the research population. Some programmatic research and public health training was administered in English, which all field staff understood and spoke fluently. Professional mentoring and coaching of case managers was in place throughout the period of the CM program by the intervention specialist. 

### 2.4. Data Collection and Analysis

From January 2009 to June 2011, data were collected by interview at baseline, and at 6, 12, and 18 month time points after participants entered CM. Data collection was completed by the same staff members that were providing CM. There were only four staff members providing CM and collecting data from the women. The maximum caseload was 31 women at any one time. Women worked with the same staff member for the duration of CM except for eight women who transferred from one staff member to another due to staff turnover.

Outcomes of interest included rates of alcohol consumption and a measure of well-being or contentment both during and in the months after their pregnancy. Items used to assess outcomes over time included independent measures of quantity, frequency, timing and context of drinking, such as the number of drinks consumed per day, per week, and on weekends. This was captured with a seven-day recall measurement of alcohol consumption. Women were asked at each 6-month follow-up interview to report their alcohol consumption for each day of the past week. In addition, women were asked at their baseline visit to recall a typical week of drinking three months prior to pregnancy and during each trimester up to their current trimester. Therefore, if a woman entered the study in her second trimester, as most women did, she would be asked to provide a seven-day recall of alcohol for the past week, a typical week three months before pregnancy, a typical week in the first trimester, and a typical week in the second trimester. The drinking problem scale used in this evaluation was the AUDIT. For analysis, individuals in CM were separated into two risk groups based on a baseline AUDIT cut-off score of 20, which identifies those with more high-risk, dependent drinking [14]. There were 34 women (50.7%) in the high risk alcohol group and 33 women (49.3%) in the moderate risk alcohol group.

Participant’s well-being and contentment was assessed through the use of the adult Happiness Scale [20,21], which consists of ten life domains including alcohol and drug use, money management, emotional life, and marriage and family relationships. Three additional domains were added including cultural life, general happiness, and drug use as a separate category from alcohol/sobriety, for a total of thirteen life domains. Happiness is rated in each domain using a 10-point Likert scale and the total happiness measure is a sum of responses from all domains with a total score of 130 points possible if individuals are completely happy in all thirteen domains. A 10-point scale emoticon was used to administer the Happiness Scale in order to make it more understandable for the target population. For analysis, individuals in CM were separated into three happiness groups based on their scores at baseline. Women with total happiness scores from 52 to 85 were labeled as low (*n* = 22, 32.8%), women with scores from 86 to 104 were labeled as moderate (*n* = 24, 35.8%), and women with scores from 105 to 122 were labeled as having high happiness (*n* = 21, 31.3%). These happiness categories were defined statistically by separating the dataset in three equal groups.

Data analysis was completed using SPSS Version 22 [23]. Descriptive analyses were used to examine the sample characteristics and maternal risk characteristics. Independent samples *t*-tests were used to understand differences between those who completed CM and those who dropped out prior to 18 months. Paired samples *t*-tests were used to analyze change in reported drinking quantity before pregnancy and at each trimester. Pearson correlation coefficients were used to understand the relationship between drinking behavior on particular nights of the weekend and general happiness. Paired samples *t*-tests and Pearson correlation coefficients were analyzed for all individuals enrolled in CM if they had data at any given time point. Repeated measures analyses with Bonferroni *post hoc* testing as appropriate were completed to look at change in drinking habits and happiness levels over time. Repeated measures analysis allows for the examination of differences in individual scores over time (within subjects main effect) and for particular groups such as those with high *vs.* low AUDIT scores (between subjects effect). Additionally, it can be used to look for an interaction between time and group (within subjects effect), which shows whether the groups differ in their pattern of change over time. Repeated measures analyses are limited to those women with complete data at all 4 time points. Although 51 women completed CM, 1 individual missed a data collection time point. Therefore, the repeated measures analyses are limited to the 50 individuals who completed CM without missing any other timepoint.

## 3. Results and Discussion

### 3.1. Descriptive Statistics for the Sample

Table 1 provides an overview of maternal risk factors for all women enrolled in CM at their baseline visit. Women ranged in age from 15 to 40 years with a mean age of 24.9 years. Age of first drink ranged from 10 years of age to 22 years of age. Only 29.9% of individuals (*n* = 20) abstained from alcohol for the 30 days prior to the start of CM; however, 55.2% of women (*n* = 37) reported not having any alcohol in the past week. Given this discrepancy, it is likely that many women drank less between their screening visit and their baseline visit initiating the start of CM when weekly drinking was first assessed. For those individuals who reported drinking in the past 7 days at baseline, the mean total number of drinks was 8.8 (*SD* = 8.8), ranging from 0.7 drinks to 45.1 drinks. Weekend drinking comprised most of that drinking with mean weekend drinks equal to 8.4 (*SD* = 7.9). Given that weekend drinking is the primary driver of overall drinking, only weekend drinking will be reported in this article. 

In addition to describing the total sample, Table 1 provides details about the maternal risk profiles of women who completed CM and women who dropped out of the study. Individuals who dropped out of CM did not differ significantly from those who completed the study on a variety of measures. Independent samples *t*-tests indicated that women who dropped out of CM were similar in age, pregnancy history, size, and drinking history compared to those women who completed CM.

As portrayed in Table 2, most of the women enrolled in CM (55%) were unemployed. Only 29% worked full-time, and for occupation 52% identified as farm workers. Farm workers are employed either full-time or seasonally, with some workers living on the farms, but others living in town. This intervention took place in a rural community comprised of many low income households. Case managers reported that those with more money can afford more alcohol; however, limited resources do not limit access to alcohol completely. Half (49%) of the women reported that their life was very or extremely stressful. More than half (57%) of the women reported that most or all of their friends drink alcohol and another 28% reported some or half drink. Drinking with friends and family is an important social activity; and in many instances the norm this target population (where a person is perceived to be alcohol dependent only if they are intoxicated every day). Alcohol use in the study population is, however, not all about the consumption of alcohol. Alcohol is also used as a way to relax, to forget about their hardships and have fun with friends. To give up drinking is to give up their whole social life, their circle of friends and sometimes the only way of coping with hardship. This makes it more difficult for them to abstain from alcohol even when they are pregnant [24,25]. Fifty-nine percent of women enrolled in CM reported church attendance as never or not very often. There were no significant differences on these variables between those who completed CM and those who did not.

**Table 1:** Selected physical, childbearing, and alcohol use data at baseline for all women in case management (*N* = 67) and comparison of those who completed 18 months of case management (*n* = 51) to women who did not (*n* = 16).

Maternal Risk Variable	All Enrolled Women (*N* = 67) Mean (*SD*)	Women Who Completed CM (*n* = 51) Mean (*SD*)	Women Who Dropped out (*n* = 16) Mean (*SD*)	*t*	*p ^*
Age (years)	24.9 (5.4)	24.8 (5.7)	25.1 (5.2)	−0.174	0.862
Gravidity	2.6 (1.4)	2.6 (1.4)	2.4 (1.3)	0.377	0.707
Parity	1.3 (1.1)	1.3 (1.1)	1.2 (1.0)	0.276	0.783
Height (cm)	155.2 (6.9)	155.8 (7.2)	153.0 (5.7)	1.427	0.158
Weight (kg)	57.7 (12.6)	58.5 (13.5)	55.1 (9.1)	0.938	0.352
Head circumference (cm)	54.5 (2.3)	54.8 (2.3)	53.6 (2.2)	1.847	0.069
BMI	23.7 (3.9)	23.8 (4.1)	23.5 (3.2)	0.259	0.797
Age of first drinking	16.0 (2.2)	16.1 (2.1)	15.9 (2.7)	0.285	0.776
Number of years drinking regularly	7.7 (4.6)	7.6 (4.8)	8.1 (4.0)	−0.424	0.673
AUDIT score (baseline)	18.9 (8.0)	18.7 (8.1)	19.6 (8.0)	−0.387	0.700

^ *p*-values compare women who completed CM *vs.* dropouts only.

**Table 2:** Selected social maternal risk factors at baseline for all women enrolled in case management (*N* = 67) and comparison of those who completed 18 months of case management (*n* = 51) to women who did not (*n* = 16).

	All Enrolled Women (*N* = 67) %	Women Who Completed CM (*n* = 51) %	Women Who Dropped out (*n* = 16) %	χ^2^	*p* ^^^
Paid work	38.8	37.3	43.8	0.216	0.642
Occupation	52.2	45.1	75.0	5.288	0.259
Farm worker	10.4	13.7	0.0		
Factory worker	10.4	11.8	6.3		
Housewife	11.9	13.7	6.3		
Other occupation	14.9	15.7	12.5		
Usually does not work	52.2	45.1	75.0		
Usual employment status					
Full-time	28.8	28.0	31.3	2.725	0.436
Part-time	6.1	8.0	0.0		
Seasonal	10.6	8.0	18.8		
Unemployed	54.5	56.0	50.0		
How stressful is life?					
Very or extremely	49.3	47.1	56.3	1.868	0.393
Somewhat or medium	25.4	23.5	31.3		
Not at all	25.4	29.4	12.5		
How many current friends drink alcohol?					
Most or all	55.2	56.9	50.0	0.502	0.918
Some or half	28.4	27.5	31.3		
None	7.5	7.8	6.3		
Has no friends	9.0	7.8	12.5		
Frequency of church attendance					
Never or not very often	59.4	60.4	56.3	0.086	0.769
Often or very often	40.6	39.6	43.8		

^ *p*-values compare women who completed CM *vs.* dropouts only.

### 3.2. Drinking Outcomes

At baseline, all women in the study were pregnant and all but six women had delivered babies by their six month follow-up appointment. At the 12 month follow-up, one individual was pregnant with another child and at 18 months, none of the women still in CM were pregnant. Mean total weekend drinks at baseline for the entire sample (*n* = 67) was 4.1 (*SD* = 7.8) drinks, which decreased slightly at six months to 3.3 (*SD* = 6.2) before increasing to 5.2 (*SD* = 10.1) at 12 months and 8.3 (*SD* = 15.8) drinks at 18 months. Overall, women enrolled in CM decrease their drinking six months into CM. However, on average, the alcohol consumption rises to levels higher than baseline at 12 and 18 months. This increase occurs after the vulnerable baby has been born.

**Figure 1:**
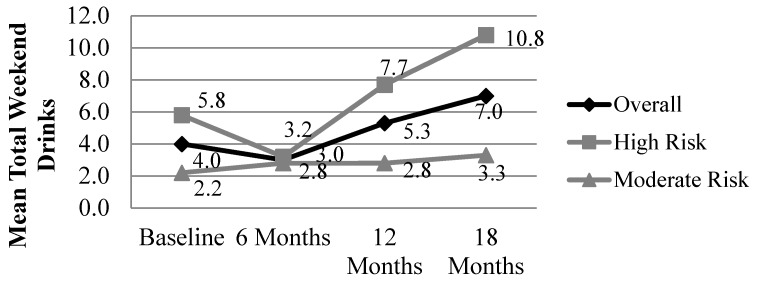
Total number of drinks consumed over the previous weekend at baseline and at 6, 12, and 18 month follow-up for women with data for all four time points (*n* = 50). Risk group determined at baseline using a cutoff AUDIT score of 20 or higher to identify women with high-risk, dependent drinking; Repeated measures analysis, within-*Ss* effect, time: *F* = 5.037, *p* = 0.029; Repeated measures analysis, within-*Ss* effect, time x risk group: *F* = 2.573, *p* = 0.115; Repeated measures analysis, between-*Ss* effect, risk group: *F* = 5.758, *p* = 0.020.

Repeated measures analyses of total drinks consumed over a weekend for only those women who completed CM (*n* = 50, see Figure 1) show a significant within-subjects main effect for time (*F* = 5.037, *p* = 0.029) and a significant between-subjects effect for risk group (*F* = 5.758, *p* = 0.020). There was a significant difference in weekend drinks at 18 months for individuals who are high-risk, dependent drinkers at baseline compared to those who report heavy drinking with fewer signs of dependency (*F* = 4.996, *p* = 0.030). The differences at baseline (*F* = 2.977, *p* = 0.091) and 12 months (*F* = 3.171, *p* = 0.081) trended towards significance, but did not meet the cutoff threshold. *Post hoc* analyses with Bonferroni adjustments for multiple comparisons did not show significant differences in drinking for specific sets of time points (*i.e.*, drinking at baseline *vs.* twelve months). 

Although most women entered CM in their second semester, we examined whether trimester was a significant measure for differentiating CM results. Repeated measures analysis of weekend drinking for women who completed CM showed no between-subjects effect for women based on trimester at the time of their baseline visit, *F* = 1.507, *p* = 0.232 or within-subjects effect for the interaction between time and trimester, *F* = 1.286, *p* = 0.286. However, the main within-subjects effect of time remained significant in this analysis, *F* = 6.439, *p* = 0.015, consistent with the results reported in Figure 1. This analysis shows that mean drinking for women in CM changed over time, but there was no difference in drinking pattern based on women’s trimester at entry into CM.

**Figure 2:**
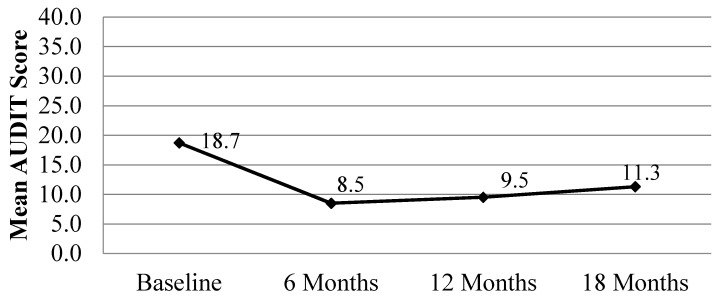
AUDIT score at baseline, 6, 12, and 18 month follow-up for women with data for all four time points. Repeated measures analysis, within-Ss effect, time: *F* = 23.323, *p* = 0.000; Pairwise comparisons (Bonferroni): Baseline *vs.* 6 month follow-up: *p* = 0.000; Baseline *vs.* 12 month follow-up: *p* = 0.000; Baseline *vs.* 18 month follow-up: *p* = 0.000.

The AUDIT captures a more comprehensive set of drinking indicators than the seven-day drinking recall reported in Figure 1. In Figure 2 it is evident that AUDIT scores decrease significantly over time for women who completed case management (*F* = 23.323, *p* = 0.000). Comparisons between baseline AUDIT scores and scores at 6, 12, and 18 months show significantly higher AUDIT scores at baseline compared to each follow-up time point. One of the most beneficial outcomes that women experienced during CM was an overall reduction in problematic drinking. 

**Figure 3:**
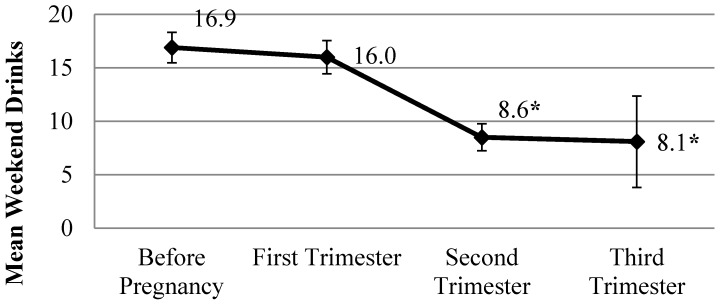
Typical weekend drinks during pregnancy reported at baseline. * *p* < 0.001; Paired samples *t*-test score comparing number of drinks consumed per weekend prior to pregnancy *versus* number of drinks consumed per weekend during each trimester.

Based on self-report at their baseline visit, women started to reduce their drinking after learning that they were pregnant and even before they entered CM. The mean number of weekend drinks for all women prior to pregnancy was 16.9 drinks with comparable weekend drinking reported in the first trimester (16.0). Weekend alcohol consumption drops significantly from before pregnancy to the second trimester (8.6 drinks), paired *t*(51) = 5.104, *p* < 0.001, and from before pregnancy to the third trimester (8.1 drinks), paired *t*(9) = 6.006, *p* < 0.001 (Figure 3). 

### 3.3. Happiness Outcomes

**Figure 4:**
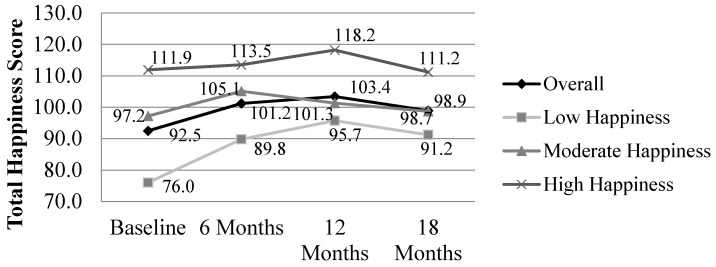
Total happiness score for women who completed case management (*n* = 50). Repeated measures analysis, within-*Ss* effect, time: *F* = 6.148, *p* = 0.001; Repeated measures analysis, within-*Ss* effect, time x group: *F* = 2.302, *p* = 0.038; Repeated measures analysis, between-*Ss* effect, group; *F* = 21.726, *p* = 0.000.

In addition to drinking outcomes, happiness was measured for all women at baseline and each follow-up time point. At baseline, women reported overall that they were the happiest in the following life domains: general happiness ($\bar{x}$ = 8.2), religion or spiritual life ($\bar{x}$ = 7.9), legal issues ($\bar{x}$ = 8.1), and their current drug use or non-use ($\bar{x}$ = 8.9). Individual life domains with the lowest mean level of happiness for all enrolled women included drinking/sobriety ($\bar{x}$ = 6.0), money management ($\bar{x}$ = 6.3), and job or educational progress ($\bar{x}$ = 6.7). An overall happiness score was evaluated for women completing CM and showed an increase from 92.5 out of a possible 130 at baseline to 101.2 at 6 months, 101.3 at 12 months, and then a slight reduction to 96.9 at 18 months as shown in Figure 4. Overall, there was a general level of happiness; however, drinking was the strongest area bringing down the total happiness score among these women at baseline.

To further understand women’s level of happiness, individual scores were statistically trichotomized and labelled as low, moderate, or high happiness. Repeated measures analysis of happiness were significant by time (*F* = 6.148, *p* = 0.001), happiness group (*F* = 21.726, *p* = 0.000), and time x happiness group interaction (*F* = 2.302, *p* = 0.038) for women who completed CM (*n* = 50). Overall, happiness increases significantly for women from baseline to 6 months (*p* = 0.002) and 12 months (*p* = 0.001) before returning to near baseline levels at 18 months (*p* = 0.323). Individuals with low levels of happiness at baseline increased their level of happiness the most during CM; however those with the highest reported happiness at baseline also increased their level of happiness at 6 and 12 months. 

Happiness is correlated with drinking quantity as displayed in Table 3. Higher total happiness scores are significantly correlated with a lower number of drinks for all Friday and Saturday time points as well as Sunday drinks at 12 and 18 months (see Table 3). The highest correlations between happiness and lower levels of drinking are at 12 and 18 month follow-up periods. These analyses include all women with data at each timepoint.

**Table 3:** Correlations between general happiness and drinking quantities on each day of the weekend.

Time Point	Friday Drinks *R* (*p*)	Saturday Drinks *R* (*p*)	Sunday Drinks *R* (*p*)
Baseline: Total happiness score	−0.242 (0.049) *	−0.369 (0.002) *	−0.154 (0.213)
6 Month: Total happiness score	−0.335 (0.008) *	−0.446 (0.000) *	−0.130 (0.319)
12 Month: Total happiness score	−0.477 (0.000) *	−0.453 (0.001) *	−0.336 (0.012) *
18 Month: Total happiness score	−0.454 (0.001) *	−0.474 (0.001) *	−0.503 (0.000) *

* *p* < 0.05.

**Figure 5:**
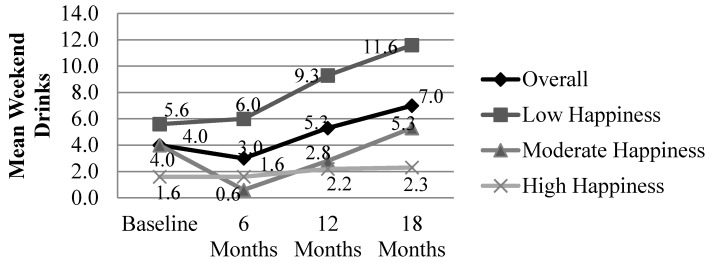
Total happiness score and weekend drinking at baseline, 6, 12, and 18 months. Data include only those women pregnant at baseline who have data for all four time periods (*n* = 50); Repeated measures analysis, within-*Ss* effect, time: *F* = 3.784, *p* = 0.058; Repeated measures analysis, within-*Ss* effect, time x group: *F* = 1.288, *p* = 0.285; Repeated measures analysis, between-*Ss* effect, group; *F* = 5.589, *p* = 0.007.

For women who completed CM (*n* = 50), repeated measures analyses of total happiness score and number of weekend drinks did not show significant differences by time (*F* = 2.185, *p* = 0.099) or time x happiness group interaction (*F* = 0.499, *p* = 0.792). However, results were significant based on women’s happiness group (*F* = 5.343, *p* = 0.008) indicating that those with low, moderate, and high levels of happiness showed different levels of weekend drinking. Those with the highest levels of happiness have the lowest levels of drinking at baseline and maintain the lowest levels of weekend drinks at 12 and 18 months. Comparatively, those with the lowest levels of happiness are the heaviest drinkers at baseline and all follow-up time points. Figure 5 portrays the lower levels of happiness associated with higher levels of weekend drinking at baseline and higher levels of happiness for those with the lowest levels of weekend drinking at baseline and throughout the duration of CM.

## 4. Discussion

### 4.1. Summary of Main Findings

Women’s alcohol consumption decreases significantly from the amount reported before pregnancy to the amount reported in the second and third trimester as women learn that they are pregnant and take action to reduce their drinking levels during pregnancy. Drinking overall persists throughout many of the pregnancies and a reduction in alcohol during pregnancy occurs only after the pregnancy is confirmed by the antenatal clinic, which is usually not until the second trimester. Mean alcohol consumption for those in the second trimester at baseline was 8.6 drinks per weekend. Compared to results previously reported [12], repeated measures analyses of weekend drinks for the entire sample of women who completed CM shows a significant change over time whereas previously this was not found. Enrolment in CM correlated with an additional alcohol reduction during the remainder of their pregnancy and the months after birth. For women who completed CM, weekend drinking dropped from 4.0 drinks at entry into CM to 3.0 drinks at the six month follow-up before surpassing baseline drinking at the 12 and 18 month follow-up. Drinking levels for women at the six month follow-up are about one-half of what has been reported for ZA women not in CM. Additionally, mean drinking levels at 12 and 18 months are lower than previous reports for women in these communities when not in CM [11]. 

More promising, AUDIT scores decrease significantly from baseline to 6 months and remain low throughout CM. This is an indication that problematic drinking may be reduced by CM and that CM could provide a major vehicle for tertiary prevention of drinking during pregnancy among these high-risk women who drink heavily in ZA. In addition to outcomes related to drinking, CM also appears to result in higher levels of happiness especially for women with the lowest levels of happiness at baseline. Overall, happiness is significantly correlated with reduced weekend drinking, especially on Friday and Saturday. This analysis of happiness and drinking by level of level of happiness at baseline is a valuable contribution that was not previously reported in the first paper about this study [12].

### 4.2. Strengths and Limitations

This was a prospective study aimed at decreasing drinking among high-risk, pregnant women. Despite high rates of social support for and practices of alcohol consumption and binge drinking in the overall project, 67 women decided to enroll in this study with the goal of reducing alcohol consumption. Most of the women had consumed alcohol after becoming pregnant, therefore, exposing their unborn fetus to alcohol in utero. The mean weekend drink quantity was 16.0 in the first trimester. Alcohol consumption during pregnancy declined, which results in better chances that their baby will not be born with FASD. Unfortunately, women did not reduce their drinking entirely even during pregnancy. Another data collection point during the third trimester for all women regardless of pregnancy stage at enrollment would have been important for fully understanding any drop in drinking during the final months of pregnancy. It is possible that drinking during pregnancy dropped more than we were able to capture at the six-month follow-up, which most often occurred in the months post-delivery. One consideration for future alcohol data collection during CM is a diary method in order to capture more complete and nuanced data about women’s drinking patterns in real time during their pregnancy.

Twenty-four percent of women enrolled in CM dropped out prior to the 18 month follow-up appointment. Women who dropped out were not statistically different from women who remained in the study at the time of their baseline study. However, their drinking and well-being outcomes experienced as part of their brief CM could not be evaluated. Additionally, women were not randomly assigned to either treatment or a control condition because the overall goal was to provide the maximum amount of benefit to the maximum number of mothers and unborn babies to help this community cope with the tremendous FASD problem uncovered by previous research. It was both ethical and practical to evaluate efficacy through change over time; however random assignment could have been made and results evaluated by case control methods. Studies in the future could benefit from collection of drinking data for a matched control group. 

The intervention offered as part of this study was individualized for each woman enrolled and targeted her specific needs for changing her drinking behaviors. Overall, CM was based on MI principles. MI offers support that is nonthreatening and shifts the focus from feelings of guilt and shame to empowerment. Women are able to make changes in their behavior with the support of the case manager; however, it was also important for the case managers to set boundaries for their involvement in order to allow women the opportunity and responsibility to make the desired changes. The amount of case manager support differed based on the needs of the enrolled women and the amount of interaction allowed was not restricted during the study. Some women received more support than other women, however, the exact amount was not tracked closely. Future evaluation of best practices in CM could benefit from a more structured CM model that maintains flexibility to allow for individual differences. This would allow for the evaluation of the level of CM engagement most beneficial for women. Additionally, it would be helpful for understanding whether women who are high-risk drinkers during pregnancy need a different level of CM engagement than those who are moderate-risk drinkers. 

### 4.3. Implications for Practice

Living circumstances, socio-economic realities, a lack of resources, and an institutionalized recreational drinking culture contribute to the difficulty that heavy drinking ZA women have to endure during pregnancy. Yet, this indicated prevention CM model built on principles of MI and CRA to effectively reduce drinking levels from baseline to six months and significantly lower AUDIT scores of problematic drinking. CM, as employed in this study, is designed as one component of an overall IOM prevention model aimed at reducing the prevalence rate of FASD over time. Focusing on indicated prevention is effective at reducing the alcohol consumed by women at high risk of having a child exposed to alcohol in utero. It is beneficial that the heaviest drinkers were actually the women who decreased their drinking the most during their pregnancy. The impact of this reduction will be seen most immediately with the current child, but also can be instrumental in future child births and with other members of their social network if they are to get pregnant. 

CM supports women in their decision to drink fewer alcoholic beverages during pregnancy, but continuation of this support and encouragement is not as effective after 6 months and the birth of the target child. Once their baby is born, the goal of abstinence from alcohol competes with the institutional pattern of recreational drinking and is no longer as effective of a deterrent. When CM is continued after their baby is born, women may need additional motivators to keep drinking down post-delivery. One possibility for engagement post-delivery is family planning for future FASD prevention, especially among women who resume their high risk drinking after the birth of a child. For many women, CM is one of the few opportunities women have to talk about their life with someone else. Many women are deserted by their husband or partner during the pregnancy. Some men move out, some find a new partner, and many drink heavily during the woman’s pregnancy [10]. The case manager is very often the only person who supports these women, believes in them, and has ever listened to them to make them feel worthy. This interaction is a key reason that CM is successful with this population as can be seen with the outcome of increased happiness throughout CM. It is important to develop additional drinking deterrents post-delivery building on this relationship if the goal is continued reduction in drinking during the child’s first year of life.

Recruiting women to CM earlier is difficult in this population, but it would greatly enhance child outcomes. Many of the pregnancies to ZA women are unplanned, so women only realize they are pregnant at a late stage in the pregnancy. Many women do not seek antenatal care until the second or third trimester and by that point, the fetus has already been exposed to large quantities of alcohol when the fetus is very vulnerable to disruption and malformation. The Department of Health in ZA has set a goal to get women to the antenatal clinics at 14 weeks instead of the 20 weeks currently, which has the benefit of confirming the pregnancy sooner and impacting pregnancy drinking sooner. Despite a culture of binge drinking, women do start to take measures to reduce drinking once they find out they are pregnant. Women who know they are pregnant begin to reduce their drinking and their decision to enter CM results in further drinking reduction in CM even as early as the screening appointment. The earlier in the pregnancy that a woman is enrolled in CM, the greater the potential benefit to her baby from months of abstinence or at least a reduction in drinking.

The use of lay counsellors at clinics who are trained to provide MI for CM that starts at the first antenatal visit could potentially impact drinking at the earliest possible point in the pregnancy. In ZA antenatal clinics, there are lay counsellors who complete counselling for HIV and other health conditions. An expansion to include CM to reduce alcohol consumption could be a valuable and logical way to make this a sustainable service in the future. The use of MI with CM is not time consuming; yet, it can result in positive outcomes for women during pregnancy and it is likely that positive outcomes could also be achieved in other service organizations. The use of MI in CM is also an ideal strategy to follow in welfare organizations and clinics where time is a crucial factor. Although there are costs involved with the training and provision of CM by lay counsellors, these costs would be offset by the cost savings resulting from preventing a child born with FASD and their utilization of services in the school and health systems and the fact that many end up repeating a year or two at school [7,26].

## 5. Conclusions

CM is correlated with a successful reduction in weekend drinks and less problematic drinking. This CM model provides support and encouragement for a pregnant woman with high-risk drinking behavior, which helps women drink less during the initial time frame of the intervention. If the intervention is effective, consequently, women reduce their risk of giving birth to a child with FASD or reduce the severity of associated disabilities. Increasing individual levels of happiness appears to be a major correlate of reduced drinking among these high-risk pregnant women and an important outcome of the study. Women who are the least happy, drink the most. Happiness increases when they drink less while pregnant and decreases again when they start drinking more. CM as an indicated prevention method should be continued in combination with selective and universal prevention methods in order to reduce the prevalence of FASD in this community. Additionally, this model of CM could be utilized in other communities with high-risk mothers in order to reduce the prevalence of FASD in those communities.
